# A Wearable Mixed Reality Platform to Augment Overground Walking: A Feasibility Study

**DOI:** 10.3389/fnhum.2022.868074

**Published:** 2022-06-09

**Authors:** Emily Evans, Megan Dass, William M. Muter, Christopher Tuthill, Andrew Q. Tan, Randy D. Trumbower

**Affiliations:** ^1^Spaulding Rehabilitation Hospital, Cambridge, MA, United States; ^2^Department of Physical Medicine and Rehabilitation, Harvard Medical School, Boston, MA, United States; ^3^Georgia Institute of Technology, School of Computer Science, Atlanta, GA, United States; ^4^Department of Integrative Physiology, University of Colorado Boulder, Boulder, CO, United States

**Keywords:** motor learning and control, walking, mixed reality, visual feedback, rehabilitation, kinematics

## Abstract

Humans routinely modify their walking speed to adapt to functional goals and physical demands. However, damage to the central nervous system (CNS) often results in abnormal modulation of walking speed and increased risk of falls. There is considerable interest in treatment modalities that can provide safe and salient training opportunities, feedback about walking performance, and that may augment less reliable sensory feedback within the CNS after injury or disease. Fully immersive virtual reality technologies show benefits in boosting training-related gains in walking performance; however, they lack views of the real world that may limit functional carryover. Augmented reality and mixed reality head-mount displays (MR-HMD) provide partially immersive environments to extend the virtual reality benefits of interacting with virtual objects but within an unobstructed view of the real world. Despite this potential advantage, the feasibility of using MR-HMD visual feedback to promote goal-directed changes in overground walking speed remains unclear. Thus, we developed and evaluated a novel mixed reality application using the Microsoft HoloLens MR-HMD that provided real-time walking speed targets and augmented visual feedback during overground walking. We tested the application in a group of adults not living with disability and examined if they could use the targets and visual feedback to walk at 85%, 100%, and 115% of each individual’s self-selected speed. We examined whether individuals were able to meet each target gait speed and explored differences in accuracy across repeated trials and at the different speeds. Additionally, given the importance of task-specificity to therapeutic interventions, we examined if walking speed adjustment strategies were consistent with those observed during usual overground walking, and if walking with the MR-HMD resulted in increased variability in gait parameters. Overall, participants matched their overground walking speed to the target speed of the MR-HMD visual feedback conditions (all *p*-values > 0.05). The percent inaccuracy was approximately 5% across all speed matching conditions and remained consistent across walking trials after the first overall walking trial. Walking with the MR-HMD did not result in more variability in walking speed, however, we observed more variability in stride length and time when walking with feedback from the MR-HMD compared to walking without feedback. The findings offer support for mixed reality-based visual feedback as a method to provoke goal-specific changes in overground walking behavior. Further studies are necessary to determine the clinical safety and efficacy of this MR-HMD technology to provide extrinsic sensory feedback in combination with traditional treatments in rehabilitation.

## Introduction

Walking speed is a predictor of functional independence, health, and mortality risk (Schmid et al., 2007; Middleton et al., 2015). Humans modify their walking to negotiate different walking terrains and obstacles (Glaister et al., 2007; Orendurff et al., 2008). These adjustments occur through regulatory networks within the central nervous system (CNS) that transform afferent information (e.g., proprioception) to motor outputs based on the interactions between the limb and the physical demands of the task (i.e., functional walking). Deficits in the sensory feedback system are known to contribute to increased incidence of walking-related falls in older adults (Afilalo et al., 2010; Artaud et al., 2015) and persons with neuromuscular injury or disease (Perry et al., 1995; Hausdorff et al., 2001; Dodge et al., 2012). There is considerable interest in treatment modalities that can provide salient training opportunities, feedback about walking performance, and may augment less reliable or weak afferent signaling to help improve walking ability and decrease risk for fall in these at-risk groups.

Studies confirm that visual feedback provided through virtual environments enhances walking ability (Schliessmann et al., 2014; Gomez-Jordana et al., 2018). Technologies that provide visual information and feedback include head-mounted displays (HMD), monitors, or large screen projectors (e.g., cave automatic virtual environment, CAVE) to create partially or fully immersive environments (Bishop and Fuchs, 1992; Milgram et al., 1995). Some studies support the use of fully immersive virtual reality (VR) for walking rehabilitation (Borrego et al., 2016; Janeh and Steinicke, 2021). These virtual environments rely on “walking-in-place” or “redirected walking” strategies to emulate features of the real world. While these gaming-related strategies provide engagement and motivation (Lohse et al., 2013), they do not fully capture the dynamic challenges of overground walking in a person’s home or community. For instance, the “walking-in-place” training programs often involve a treadmill that introduces biomechanical differences to overground walking (Dingwell et al., 2001; Lee and Hidler, 2008; Hollman et al., 2016; Ochoa et al., 2017), and this discrepancy may dampen carryover of the treadmill training to overground walking tasks. Consideration for more ecologically favorable augmented reality environments is of high value in the rehabilitation community.

Mixed reality (MR) technology is emerging as a promising approach to accommodate the real-world challenges of relearning to walk in the home or community after injury or disease. Mixed reality incorporates computer-rendered objects within an unobstructed view of the real world and enables real-time interactions with these virtual elements (O’Connell, 2016). In 2016, Microsoft introduced the HoloLens (HoloLens 1st Generation, Microsoft Inc., Redmond, WA), a head-mounted MR system that includes an inertial measurement unit, environment sensing cameras, and a holographic display. A pair of recent studies found the potential utility of the HoloLens as a gait assessment tool to track spatiotemporal gait parameters (Geerse et al., 2020; Guinet et al., 2021) reporting the system reliable at test-retest parameterization of walking speed (interclass correlation coefficient, ICC = 0.86), cadence (ICC = 0.88), and step length (ICC = 0.77). Additionally, Coolen and colleagues developed a training tool to facilitate virtual obstacle-avoidance training with the HoloLens (Coolen et al., 2020). They found the virtual obstacle avoidance task elicited lead and lag step height maneuvers similar to a physical obstacle-avoidance task during overground walking in adults living without disability. This prior work indicates that MR is achievable for assessing overground walking behavior and creating realistic environmental challenges; however, the potential of MR to induce modulation of overground walking behavior (i.e., speed) is not known.

The purpose of this study is to examine the feasibility of MR-based visual feedback to promote goal-directed changes in overground walking in preparation for future intervention studies aimed at identifying effective strategies for improving gait speed, stability, and ability to adjust walking to environmental demands. As a first step, we examine the use of a MR-HMD and a novel visual feedback platform in adults living without a known walking impairment. Specifically, we examine how accurately this group of individuals can match a target speed provided by the MR-HMD, if the accuracy changes with practice using the device or at different target speeds, if individuals use expected strategies to adjust gait speed, and if using the MR-HMD results in any additional variability in gait speed or parameters that should be considered when designing future trials. We predicted that participants would adjust their walking speed to match the visual guidance of a custom MR-HMD platform. We quantified the performance of participants interacting with the MR-HMD platform in terms of speed matching accuracy across trials and between walking speed conditions. We examined if individuals adjusted gait parameters as expected when adjusting gait speed. Finally, we compared changes and variability of walking speed parameters (i.e., stride length and time) during self-selected walking with and without the MR-based visual feedback.

## Methods

We conducted a block-randomized, cross-over intervention study to test the hypothesis that a novel MR-based visual feedback platform is a feasible method to elicit modulation of overground walking speed in adults not living with disability. The study took place at Spaulding Rehabilitation Hospital, Boston, USA.

### Participants

We enrolled 12 adults, not living with disability, to participate in the single-day study (Table 1). Eligible participants included persons between the ages of 18 and 75 years with the ability to follow two-step commands and to walk overground without assistance. We excluded individuals with uncorrected moderate to severe visual impairments, movement deficits, or neuromuscular impairments. Eligible participants meeting the inclusion/exclusion criteria provided informed consent prior to study participation. We obtained study approval from the Mass General Brigham institutional review board.

**Table 1:** Demographics.

Participant	Sex	Age (years)	Height (cm)	Weight (kg)	Self-Selected Walking Speed (m/s)
1	M	23	173	65.8	1.51
2	M	28	183	77.1	1.24
3	M	48	188	77.6	1.91
4	M	64	178	113.4	1.33
5	F	23	157	60.3	1.53
6	M	43	177	83.9	1.40
7	F	27	163	54.4	1.61
8	M	34	173	70.3	1.34
9	F	30	152	49.9	1.43
10	F	24	173	68.0	1.45
11	M	29	176	74.8	1.40
12	M	23	191	81.6	1.15
Mean ± SD	NA	33 ± 12.6	174 ± 11	73.1 ± 16.5	1.44 ± 0.19

*Note: SD, Standard deviation*.

### Equipment

#### Wearable Mixed-Reality Hardware

**Figure 1:**
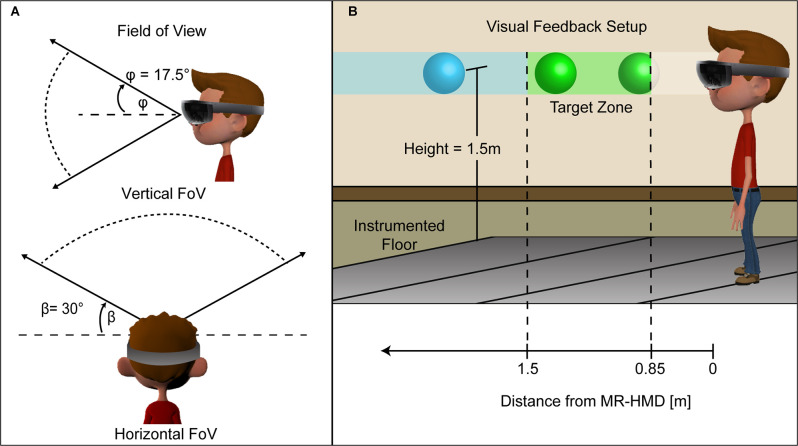
Visualization of the HoloLens MR-HMD. The image on the left **(A)** shows the vertical and horizontal field of view for rendered holographic objects. The visual feedback **(B)** consisted of a floating holographic ball in different sections based on distance from the HMD. When maintaining the target speed, the ball remains between 1.5 m and 0.85 m from the subject and is colored green. If the subject walked too slowly and the ball was beyond 1.5 m its color changed to blue providing additional feedback that the user should walk faster. The ball clipped when it was less than 0.85 m from the HMD indicating that the subject should walk more slowly.

We implemented real-time visual feedback using the Microsoft HoloLens, a commercially available MR-HMD (HoloLens 1st Generation, Model 1688, Microsoft Inc., Redmond, WA). The MR-HMD implemented the Windows Mixed Reality platform (Microsoft Inc., Redmond, WA) to allow viewing of virtual objects overlaid onto a real-world environment *via* a transparent visor having a fixed field of view (Figure 1A). The headband diameter and fore-aft location of the MR-HMD are both adjustable to ensure user comfort and visual quality. The technology included a time-of-flight depth-sensing camera, four tracking cameras, and an inertial measurement unit consisting of a multidimensional accelerometer, gyroscope, and magnetometer. The sensors enabled the MR-HMD system to define the headset’s location relative to the three-dimensional physical environment (Hubner et al., 2020). Based on these inputs, the MR-HMD system updated the location, depth, and orientation of the holographic display (Kubben and Sinlae, 2019).

#### Visual Feedback Platform

A custom MR-HMD user interface provided personalized real-time visual feedback for modulating walking speed. The interface included a moving hologram that served as the speed-matching target and the real-time feedback of the participant’s speed-matching accuracy. The visual feedback consisted of a 10 cm translucent holographic ball that moved along a 10 m path at a fixed height of 1.5 m above the ground (Figure 1B). We developed this graphical interface using a cross-platform game engine (Unity version 5.5.0f3, Unity Technologies, San Francisco, CA) and C# (Visual Studio 2017, Microsoft Inc, Redmond, WA). As the HoloLens only rendered a single holographic object to provide the continuous visual feedback the frame rate was synced to the MR-HMD refresh rate of 60 Hz to prevent tearing artifacts (Lee et al., 2017).

#### Instrumented Overground Walkway

We recorded the participants’ walking kinematics using a 14 m instrumented walkway (GAITRite CIRFace, CIR Systems Inc., Franklin, NJ). The walkway has high test-retest reliability, concurrent validity within and between systems, and used previously to assess overground walking in persons living with and without movement related disability (Thibaudier et al., 2020). We acquired the kinematic data in the sagittal plane at a sampling rate 100 Hz over a 10 m distance. Participants walked a total of 14 m that included 2 m before and after the 10 m collection area; this reduced potential confounding effects of initial and final accelerations on the walking kinematics. Using a secure desktop computer, we stored video and kinematic data for further data processing and statistical analyses.

### Experimental Protocol

We fitted each participant to the MR-HMD technology. The experiment team aided participants with donning the device and adjusting the head straps for their comfort. Participants wore their usual corrective eyewear. Once fitted, participants learned to interact with the MR interface using various simple hand gestures. This included a calibration procedure that ensured the holographic display aligned with the user’s visual field. The calibration protocol involved a Microsoft Windows Mixed Reality alignment routine to estimate the user’s interpupillary distance based on a series of finger target-matching tasks for each eye. The MR-HMD platform then incorporated the calibration transform to modify the holographic ball orientation within the real-world environment. Once calibrated, the 10 m walking course was defined over the instrumented walkway using the holographic interface. Completion of the MR-HMD fitting and calibration procedures took approximately 10 min.

The experimental protocol required participants to complete a baseline walking assessment. We instructed participants to walk at their self-selected (SS) walking speed across the instrumented walkway. During baseline trials, the MR-HMD was worn in the powered-down state such that individuals could see their environment but could not see any holographic elements. We did this to control for any passive effects of the device unrelated to visual feedback. Participants completed 10 baseline trials at their SS walking speed. This baseline assessment took approximately 10 min.

We programmed the visual feedback so that the holographic ball moved at speeds corresponding to three walking speed conditions: 85% (SS_85_), 100% (SS_100_), and 115% (SS_115_; Hayes et al., 2014) of the baseline SS without visual feedback. We chose to evaluate walking at SS speeds to understand the utility of MR-HMD during speed conditions that are relevant to community walking (Bowden et al., 2008; Talkowski et al., 2008; Stevens et al., 2013). Additionally, a variety of walking speeds were used to acquire a rich set of stepping patterns for exploring kinematic variability that represents a range of possible community walking speeds (van Hedel et al., 2006). We block-randomized the order of these walking conditions for each participant.

At the start of a walking condition, we informed participants that a holographic ball will move in the forward direction at a constant speed. To prepare participants for the ball’s movement, we implemented a visual countdown signal of the holographic ball blinking yellow four times before changing to solid green. Participants received instruction to maintain their initial distance (~1.0 m) behind the moving holographic ball. Participants remained blinded to the speed of the holographic ball during the protocol. We instructed the participants that the color of the moving ball indicated their performance. If the distance between the ball and participant stayed within the range of 0.85–1.5 m, the ball color remained green. If this distance exceeded 1.5 m, the ball color changed to blue indicating the participant needed to walk faster. If this distance did not reach 0.85 m, the ball disappeared indicating that the participant walked past the ball and needed to walk slower (Figure 1B). Participants completed ten trials at each walking condition and received rest breaks between blocks as needed to minimize fatigue. This evaluation took approximately 30 min.

### Data Processing

We quantified three overground walking parameters: walking speed, stride length, and stride time. We recorded walking speed, as the distance walked per trial divided by the duration of time to complete the trial. The stride length corresponded to the distance between the first contact of two consecutive steps of the same foot and stride time corresponded to the duration of time to complete a stride (Perry and Burnfield, 2010). Since our estimates of stride parameters did not differ between legs (Wilcoxon signed-rank test, *p*-values > 0.20), we combined left and right strides for all analyses.

We assessed the participants’ speed-matching ability using two methods. First, we calculated the percent difference in walking speed across all walking trials for a condition and examined if it was as expected for each condition, i.e., 15% slower, equal to, or 15% faster than the baseline condition. Next, to determine if the speed of the holographic ball influenced how well participants walking speed matched the ball’s speed, we calculated the percent inaccuracy for each walking trial. The percent inaccuracy is equal to the absolute percent difference between target walking speed and *actual* walking speed for each walking condition (SS_85_, SS_100_, SS_115_). Lastly, to assess step-to-step consistency in walking, we calculated the coefficient of variation (CoV), a unitless measure of variability, within individual and across walking trials for each walking parameter. The CoV was calculated for each parameter (gait speed, stride length and stride time) using the following formula CoV = σ/μ where μ is the mean value by individual and walking speed condition σ is the standard deviation by individual and walking speed condition.

### Statistical Analysis

We used Stata 17.0 (StataCorp LLC, College Station, TX) for statistical analysis. To examine the effect of practice, we tested for time-dependent changes in trial inaccuracy over the 10 walking trials of each walking condition using a linear mixed model with restricted maximum likelihood estimation. We utilized a linear mixed model to account for the repeated effects of participant, walking condition, and to allow for flexibility in the number of walking trials per condition (Davis, 2002). The model included participant random effects and a distinct covariance structure for the repeated effect of experimental conditions. We also included fixed effects of trial number, a variable reflecting if the condition was the first, second, or third tested condition, and their interaction. We calculated the marginal means values based on the interaction of trial number and condition order and conducted pairwise comparisons with Bonferroni corrections to compare the means. We hypothesized that participants may exhibit more inaccuracy during their first several trials as they learned to use the device. To avoid the potential confound of higher inaccuracy in the early trials, we planned to remove early trials, starting with the first trial until a consistent rate of accuracy was achieved. Second, to determine if our participants were able to match the target gait speed, we utilized a one-sample Wilcoxon signed-rank test to determine if the median percent difference between the target walking speed and the actual walking speed corresponded to the expected percent difference for each walking condition, i.e., −15% for SS_85_, 0% for SS_100_, and 15% for SS_115_.

Next, we utilized linear mixed models to examine percent inaccuracy and variability in gait speed, stride length, and stride time across multiple walking conditions using linear mixed models. We examined if participants ability to meet the target speed differed by walking speed by comparing the percent inaccuracy across the three tested speeds (SS_85_, SS_100_, SS_115_). Due to the importance of task-specificity in gait training interventions (Kleim and Jones, 2008), we examined if adjustments to stride length and stride time were consistent with the adjustment strategies used by healthy adults to achieve changes in overground gait speed. Specifically, healthy adults adjust stride length and stride time in opposite but equal amounts to achieve changes in gait speed (Murray et al., 1969, 1970). We examined the magnitude of the percent difference in stride length and stride time between the SS_85_ and SS_100_ conditions, and the SS_100_ and SS_115_ conditions. Because the adjustments to stride length and time are expected to be in opposite directions, we compared the additive inverse of stride length to stride time. Finally, to determine if receiving visual feedback from the MR-HMD induced additional variability in gait speed or the associated parameters, we compared the CoV of stride length, stride time, and gait speed between the baseline condition (Self-Selected Speed, MR-HMD turned off) and the SS_100_ (Self-Selected speed, visual feedback from the MR-HMD.).

Each model included participant random effects and a distinct covariance structure for the repeated effect of experimental condition. The design for modeling residuals and the between-subject covariance structure was determined by comparing Akaike’s information criterion (AIC) and Bayesian information criterion (BIC) between models (Akaike, 1974). For each model, we calculated marginal means and utilized pairwise comparisons with Bonferroni corrections to compare the marginal effects between experimental conditions. Specifically, to examine the effect of the MR-HMD feedback at the same speed, we compared marginal effects between the baseline and SS_100_ experimental conditions. We also examined the effect of walking speed changes by comparing the adjusted mean values between the experimental conditions (SS_85_, SS_100_, SS_115_). The significance of any interactions was examined using an omnibus Wald test. We reported significant results at the *p* < 0.05 level.

## Results

**Figure 2:**
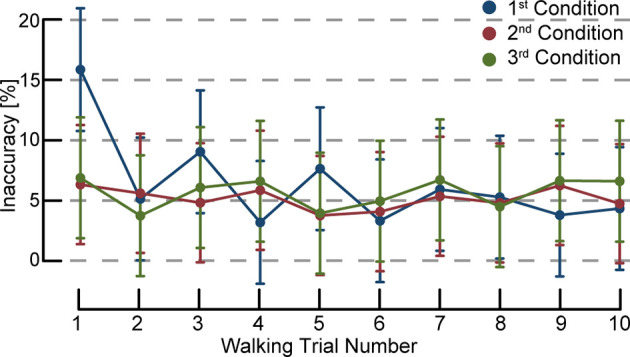
Walking speed inaccuracy percentage by trial number. The first condition using visual feedback is indicated in blue, the second tested condition in red, and third tested condition in green. Error bars denote 95% confidence intervals. The first overall trial regardless of speed condition had a larger percent inaccuracy compared with nearly all subsequent walking trials. The within-trial inaccuracy was 10.5% higher (CI95: 7.4, 13.5) in first trial of the first speed condition compared to subsequent trials. We found no significant differences in walking speed inaccuracy between the second and 10th trial of the first speed condition, or the 1st and 10th trial in the subsequent speed conditions. As the first trial overall appears to be an outlier due to a learning effect, we excluded that trial for the subsequent analyses.

Participants adjusted their walking speed to meet the target walking speed during the MR-HMD walking conditions. As expected, inaccuracy in meeting the target gait speed decreased with additional repetitions using the MR-HMD. Specifically, we found a significant interaction between within-condition walking trial number (1–10) and the order in which the condition was tested (first, second, or third; χ^2^ = 39.3, *p* < 0.01). The first trial in any condition had a larger percent inaccuracy compared with nearly all subsequent walking trials, but only if the condition was the first tested condition overall. The exception was the third trial of the first tested condition which did not differ significantly from the first trial of the first test condition (difference: −6.8%, CI95: −15.1, 1.41). Figure 2 illustrates the percent inaccuracy across the 10 walking trials in the first, second, and third tested condition. trials. Overall, the within-trial inaccuracy was 10.5% higher (CI95: 7.4, 13.5) in first trial of the first speed condition compared to subsequent trials. We found no significant differences in walking speed inaccuracy between the 2nd and 10th trial of the first speed condition, or the 1st and 10th trial in the second and third tested condition. Due to the significantly higher percent inaccuracy in the first overall walking trial, we excluded the first trial with the MR-HMD of the 30 walking trials with the MR-HMD for the subsequent analyses. We included overall walking trial number 2 through 30 for a total of 29 total walking trials. In effect, the first overall walking trial became a “practice trial”, and for each participant we analyzed nine trials of their first tested condition, and 10 walking trials each of the second and third tested condition.

**Figure 3:**
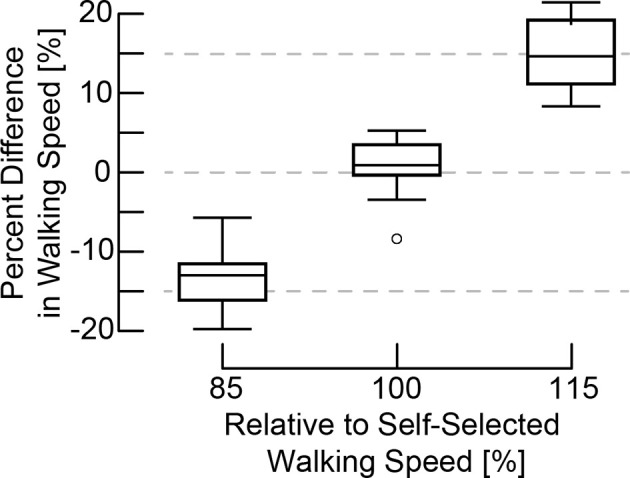
Percent difference in walking speed: actual vs. target. A one-sample Wilcoxon signed-rank test found that actual walking speeds of the participants did not differ from the expected speeds during the SS_85_ (median difference from baseline: −13.8%, *z* = 0.63, *p* = 0.53), SS_100_ (median difference from 1.2%, *z* = 1.23, *p* = 0.23), and SS_115_ (median difference: 14.2%, *z* = 0.16, *p* = 0.88) conditions.

Participants changed their overground walking speed, stride length, and stride time to adhere to the MR-HMD feedback conditions. Results of the one-sample Wilcoxon signed-rank test found that, overall, actual walking speeds of the participants did not differ from the expected speeds during the SS_85_ (median difference from baseline: −13.8%, *z* = 0.63, *p* = 0.53), SS_100_ (median difference from baseline 1.2%, *z* = 1.23, *p* = 0.21), and SS_115_ (median difference: 14.2%, *z* = 0.16, *p* = 0.88) conditions (Figure 3). Overall, the within trial inaccuracy was 5.3% (CI95: 4.3, 6.4) which was consistent across the experimental conditions (see Table 2). When using the MR-HMD, participants adjusted their stride length and stride time relatively equally when modulating gait speed. In the SS_85_ condition, stride length was shorter (−7.6%, CI95: −10.9, −4.3) and stride time was longer (7.5%, CI95: 4.2, 10.8) compared to the S_100_ condition indicating that individuals took shorter and slower steps to decrease walking speed. Applying the additive inverse of stride time, we found no difference in the magnitude of the percent change between the stride parameters (difference: 0.1%, CI95: −3.6, 3.4). Stride length was longer (7.9, CI95: 4.4, 11.4) and stride time was shorter (−5.0, CI95: −8.5, −1.5) during the SS_115_ condition as compared to the SS_100_ condition, indicating that individuals took longer and faster steps to increase walking speed. We found no significant difference in the magnitude of the change (difference: 2.8%, CI95: 0.86). However, stride length (−3.1%, CI95: −5.0, −1.2) and stride time (−3.5%, CI95: −5.3, −1.6) were both shorter when walking with feedback compared to walking without feedback at a self-selected speed.

**Table 2:** Inaccuracy percentage for each walking speed condition using the MR-HMD.

Walking speed condition	% Inaccuracy (CI95)	Comparison	Difference in % inaccuracy (CI95)
SS_85_	5.0 (3.8, 6.2)	SS_100_ vs. SS_85_	0.2 (−1.1, 1.5)
SS_100_	5.2 (4.0, 6.4)	SS_115_ vs. SS_85_	0.9 (−0.6, 2.3)
SS_115_	5.9 (4.5, 7.2)	SS_115_ vs. SS_100_	0.7 (−0.8, 2.2)
All Conditions	5.3 (4.3, 6.4)	NA	NA

*Note: SS_85_, Target was 85% of baseline self-selected speed; SS_100_, Target was 100% of baseline self-selected speed; SS_115_, Target was 115% of baseline self-selected speed; CI95, 95% Confidence Interval; NA, Not Applicable*.

**Figure 4:**
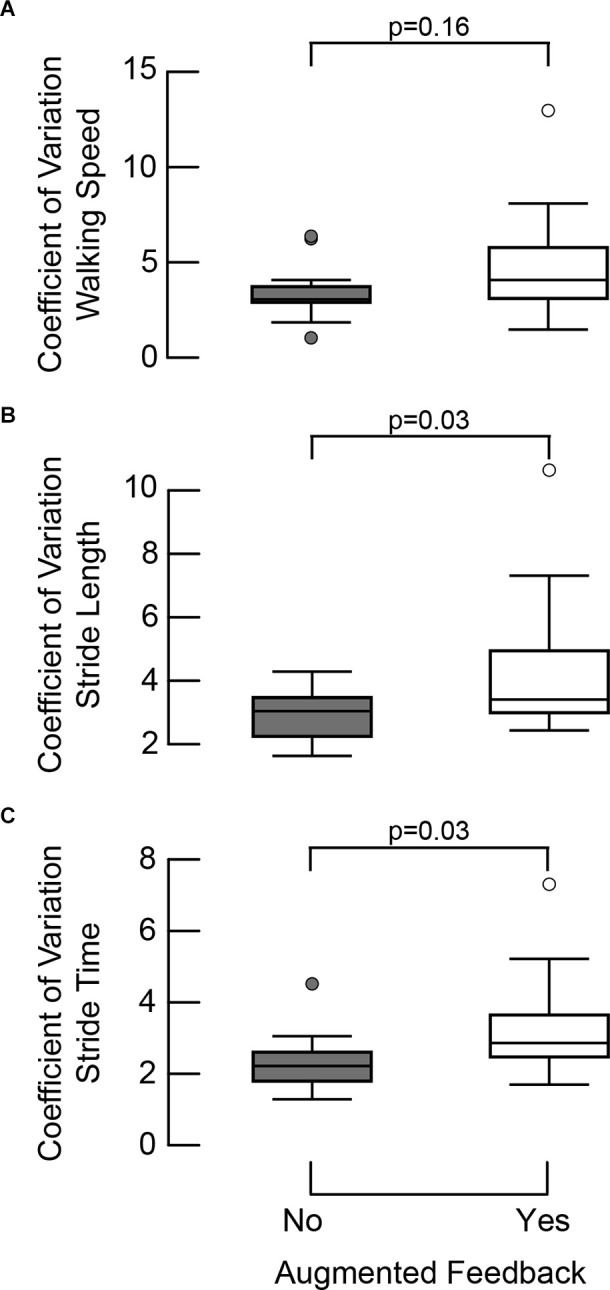
Coefficient of variation in walking speed, stride length, and stride time with and without augmented feedback. **(A)** Comparison of the baseline SS with SS_100_ condition showed that variability of walking speed did not differ when walking overground with and without MR-HMD feedback (CoV difference: 1.44, CI95: −0.54, 3.42, *p* = 0.16). **(B)** There was more variability in stride length (CoV difference: 1.47, CI95: 0.16, 2.78, *p* = 0.03) and **(C)** stride time (CoV difference: 1.00, CI95: 0.10, 1.88, *p* = 0.03) with MR-HMD feedback suggesting that individuals adopted more variable control strategies to achieve the target speed.

Variability of SS walking speed did not differ between overground walking with and without MR-HMD feedback (CoV difference: 1.44, CI95: −0.54, 3.42, *p* = 0.16; Figure 4A). However, we found more variability in stride length (CoV difference: 1.47, CI95: 0.16, 2.78, *p* = 0.03) and stride time (CoV difference: 1.00, CI95: 0.11, 1.88, *p* = 0.03) with MR-HMD feedback as compared to without MR-HMD feedback (Figures 4B,C). Our findings suggests that an individual’s walking speed was no more variable with MR-HMD feedback compared to HMD without feedback; however, individuals exhibited more variability in the parameters used to achieve the target speed. The variability of each gait parameter was consistent across speeds (Table 3). Our participants had no episodes of falls or tripping during the study and did not report any adverse symptoms during the walking trials.

**Table 3:** Difference in coefficient of variation between target walking speeds with visual feedback.

	Gait speed		Stride length		Stride time	
Comparison	Difference CI95	*p*-value	Difference CI95	*p*-value	Difference CI95	*p*-value
SS_100_ vs. SS_85_	0.73 (−1.82, 3.28)	0.99	−0.15 (−1.82, 1.52)	0.99	−0.15 (−1.82, 1.52)	0.99
SS_115_ vs. SS_85_	1.89 (−0.66, 4.45)	0.23	−0.11 (−1.34, 1.12)	0.99	0.78 (−0.56, 2.13)	0.50
SS_115_ vs. SS_100_	1.17 (−1.91, 4.25)	0.99	0.04 (−1.75, 1.83)	0.99	0.93 (−0.52, 2.37)	0.37

*Note: SS_85_, Target was 85% of baseline self-selected speed; SS_100_, Target was 100% of baseline self-selected speed; SS_115_, Target was 115% of baseline self-selected speed*.

## Discussion

This study examined the feasibility of using an MR-HMD platform to elicit goal-directed changes in overground walking. In particular, we assessed the utility of a commercially available MR-HMD technology, the Microsoft HoloLens, to promote real-time modulation of walking speed in a group of adults who are not living with disability. Participants matched overground walking speeds within approximately 5% of the MR-HMD visual feedback target speed conditions, equivalent to an average difference of 0.08 m/s. This difference is less than 0.1–0.2 m/s, a threshold considered to represent meaningful change in clinical populations (Bohannon and Glenney, 2014; Bohannon and Wang, 2019). The relatively low inaccuracy indicates that the MR-HMD is a viable tool to provoke real-time changes in overground walking behavior. We discuss these findings in the context of motor learning principles and clinical applications. Future research may extend our understanding of ways to incorporate MR platforms as adjuvants to gait rehabilitation.

### Augmented Feedback and Motor Learning

Augmented feedback has been shown to improve the acquisition of motor skills during interactions with real and virtual objects (Schmidt and Wrisberg, 2004). The type and timing of augmented feedback can be varied to enhance motor learning. For example, feedback can be internally focused, e.g., specific joint kinematics, or externally focused on completion of the full task (Wulf and Dufek, 2009). The timing of feedback can also be varied, for example, provided concurrently (during task performance), or terminally (after task completion; Sigrist et al., 2013). Additionally, augmented feedback can be provided by visual, auditory, or haptic inputs or combination multisensory modalities. In this study, our participants appeared to successfully modulate their gait speed during a single-day session when provided concurrent visual feedback and an external focus by the MR-HMD. Our findings align with substantial evidence supporting an external focus to promote motor performance and learning (Wulf, 2013; Chua et al., 2021). Our results also align with previous research which reports the benefits of extrinsic visual cues on motor learning in healthy individuals (Todorov et al., 1997; Sigrist et al., 2013; Lewthwaite and Wulf, 2017) and persons with physical disability (Aung and Al-Jumaily, 2014). Additionally, our results align with prior studies that show continuous visual cues of a movement-related task enhances the immediate learning of that task (Winstein and Schmidt, 1990; Winstein et al., 1996; Todorov et al., 1997; Weeks and Kordus, 1998; van Vliet and Wulf, 2006).

However, excessive reliance on concurrent visual feedback to achieve these goals may not translate to long-term carryover (Schmidt and Wulf, 1997). High-frequency dosing of the MR feedback may impact the consolidation of other intrinsic feedback mechanisms important for modulating walking speed. For instance, reliance on visual cues may reduce reliance on vestibular and proprioceptive intrinsic signaling that also serve major roles in accurately and precisely regulating walking mechanics during speed adjustments. Additionally, whereas this study focused on visual feedback the synchronization between visual and other types of feedback may increase the sense of “presence” in virtual environments (Heeter, 1992; Slater, 2009; Borrego et al., 2016), enhance integration into the virtual word (Lenggenhager et al., 2007) and may further improve performance (Cameirão et al., 2012). Future research should consider the effects of more explicit goal-directed MR feedback that include visualizations that complement the person’s skill level, as well as kinesthetic information about the movement, and investigate longer-term impacts on MR-based training interventions.

### Clinical Implications of Mixed Reality

Restoring the ability to walk remains a highly valued goal for humans with various neuromuscular pathologies such as stroke, cerebral palsy, spinal cord injury (SCI), and Parkinson’s disease and is a major focus of rehabilitation interventions. Even small boosts in walking that promote greater independence with standing, walking within the home, or negotiating spaces not accessible with walking aides often translate into significant gains in health and quality of life. Skilled training strategies that target walking improvement should promote motor learning and neuroplastic changes *via* salient and task-specific training and effective feedback. MR platforms have the potential to enhance clinical training focused on walking improvement *via* several mechanisms (Kleim and Jones, 2008; Levin et al., 2015).

First, MR platforms embed virtual objects within the real environment thereby reducing the patient’s dependency on the simulated physical environment or the boundaries of a research laboratory. This allows for training in any environment (e.g., home, clinic, or community) that has salience to the individual. Second, MR-based interventions can provide opportunities for task-specific training. Task-specificity can reflect both the actual environment and virtual obstacles, but also the strategies used by individuals to negotiate these environments. Our findings demonstrated that while using MR-HMD individuals changed their gait speed by adjusting parameters (stride length and stride time) in the same pattern that is observed in healthy individuals during overground walking (Murray et al., 1969, 1970). Additionally, we observed similar variability in gait speed when individuals were walking with active feedback from the MR-HMD compared to walking with the powered-off device. However, we did find additional variability in stride length and stride time which suggests that using the MR-HMD may induce some variability at the individual step level.

In addition to providing salient and task specific training, MR applications have the potential to promote motivation and training adherence, and options for gamification may increase enjoyment of motor learning tasks as has been observed in VR applications (Thornton et al., 2005; Sharar et al., 2007; Burke et al., 2009; Ibrahim et al., 2016; Dias et al., 2019). MR based interventions also have the potential to improve feedback mechanisms. The provision of extrinsic forms of visual feedback regarding a physical task may enhance the performance of that task (Tate and Milner, 2010) especially when intrinsic feedback may be less reliable or inaccessible due to injury or illness (e.g., loss of proprioception).

Our findings align with previous literature supporting augmented and virtual reality as viable methods to improve rehabilitation outcomes. The use of augmented reality as an adjuvant to traditional physical therapy has been shown to enhance both trunk balance (Maciaszek et al., 2014; Cho et al., 2015) and walking performance (Yang et al., 2008; Mirelman et al., 2009; Stanton et al., 2011; Pedreira da Fonseca et al., 2017) in persons with cortical stroke. Further, the use of virtual-based feedback during treadmill walking facilitates walking in older adults (Franz et al., 2014), persons with spinal cord injury (Yen et al., 2014), and Parkinson’s disease (Jellish et al., 2015; Wang et al., 2022). These results emphasized the benefits of MR technology to enhance recovery of functional walking. The comparable effects suggest that virtual objects may be as effective as real objects at modifying stepping behaviors. Finally, a recently published clinical practice guidelines supports use of virtual reality interventions to improve locomotion for individuals with neurologic diagnoses (Hornby et al., 2020). However, many questions remain regarding dosing and timing of augmented reality experiences within the context of the traditional gait rehabilitation (Koroleva et al., 2021). This includes the management of the level of immersion (Bailenson et al., 2005; Crosbie et al., 2006) to optimize task performance while minimizing potential withdrawal effects resulting from the transition from virtual to the real world (Hughes et al., 2020).

### Study Limitations

There are several study limitations that preclude the generalization of our study findings. First, the MR-HMD hardware posed constraints on the field of view, hologram complexity, and display resolution. To accommodate the device’s limited field of view and processing power, we simplified the visual feedback experience for study participants. We programmed the holographic ball to travel along a linear 10 m path that remained at a fixed height parallel to the ground. However, functionally meaningful tasks require turns, inclines, and obstacles that require head rotations. Changes in head inertia due to the mass of the MR-HMD may result in deviations in walking task requiring head rotation, but we did not explicitly test this possibility. Differences in the variability of stride length and stride time during overground walking occurred between SS walking with the MR feedback as compared to without the feedback (HMD only), indicating that participants may have had difficulty in maintaining step-to-step kinematics during the prescribed walking speed. It should also be noted that our protocol was designed to examine immediate changes in gait speed and kinematics while individuals received feedback from the MR-HMD, but we did not examine changes to gait speed or kinematics after the device was removed or long-term adaptions to gait parameters. Future work is needed to examine the efficacy of MR feedback in individuals with and without gait impairments and may be able to leverage newer devices which could mitigate some of the limitations attributed to the technology in the 1st generation MR-HMD. The growing positive evidence of MR will inevitably lead to new challenges regarding the integration of these technologies into the traditional clinical setting (Cerritelli et al., 2021).

## Conclusions

This study demonstrated the feasibility of a novel MR-HMD platform that provided real-time visual feedback to promote goal-directed changes in overground walking speed. Participants learned to adjust their walking speed based on a single session of visual feedback from a personalized MR environment. Further advances in MR-HMD technology will no doubt result in greater accessibility and broader applications in healthcare and home settings.

## Data Availability Statement

The raw data supporting the conclusions of this article will be made available by the authors, without undue reservation.

## Ethics Statement

The studies involving human participants were reviewed and approved by Mass General Brigham Institutional Review Board. The participants provided their written informed consent to participate in this study.

## Author Contributions

EE led the analysis and manuscript writing. MD developed the mixed reality programs. WM contributed to the methods and figures. CT contributed to the methods and analysis. AT performed the experiments and contributed to the discussion. RT conceived the idea for the mixed reality programs and contributed to the introduction and discussion. All authors provided critical feedback to the manuscript. All authors contributed to the article and approved the submitted version.

## Conflict of Interest

The authors declare that the research was conducted in the absence of any commercial or financial relationships that could be construed as a potential conflict of interest.

## Publisher’s Note

All claims expressed in this article are solely those of the authors and do not necessarily represent those of their affiliated organizations, or those of the publisher, the editors and the reviewers. Any product that may be evaluated in this article, or claim that may be made by its manufacturer, is not guaranteed or endorsed by the publisher.
